# Severe cholestatic hepatitis due to large vessel vasculitis: report of two cases

**DOI:** 10.1093/gastro/gov061

**Published:** 2015-12-12

**Authors:** Jason Xu, Einar S Björnsson, Vinay Sundaram

**Affiliations:** gov061-1 Department of Medicine, Cedars-Sinai Medical Center, Los Angeles, CA, USA; gov061-2 Department of Gastroenterology and Hepatology, The National University Hospital of Iceland, Reykjavik, Iceland; gov061-3 Faculty of Medicine, University of Iceland, Reykjavik, Iceland; gov061-4 Division of Gastroenterology and Hepatology and Comprehensive Transplant Center, Cedars-Sinai Medical Center, Los Angeles, CA, USA

**Keywords:** acute liver injury, polymyalgia rheumatica, abnormal liver enzymes

## Abstract

Giant cell arteritis (GCA) is a vasculitis of medium and large sized vessels that occurs most often in people >50 years of age with associated symptoms of fever, weight loss, headache and jaw claudication. Polymyalgia rheumatica (PMR), which is characterized by aching and stiffness in the shoulders, hip girdle, neck and torso, is intimately associated with GCA, and evidence suggests that GCA and PMR are two phases of the same disease. The occurrence of liver enzyme abnormalities in either of these conditions is rare. Furthermore, as these conditions occur most commonly in the elderly population who may be subject to polypharmacy, patients with elevated aminotransferases due to underlying GCA/PMR may mistakenly have their abnormal liver function tests attributed to drug-induced liver injury. Given the potential complications of these diseases if left untreated, including ischemic stroke and blindness, early recognition and treatment are critical. We report two patients who developed severe cholestatic liver enzyme elevation, which had been initially attributed to drug toxicity, but was ultimately caused by large vessel vasculitis, specifically GCA and PMR.

## Background

Cholestatic liver enzyme elevation occurs most commonly in the setting of extrahepatic biliary obstruction or intrahepatic ductal injury. Though rare, large vessel vasculitides may present with abnormal liver function tests that primarily manifest as a rise in serum alkaline phosphatase level with normal total bilirubin. Histological changes of the liver in giant cell arteritis (GCA) and polymyalgia rheumatic (PMR) are inconsistent and nonspecific [1–4]. In most instances, hepatic enzymes normalize after adequate treatment of the underlying vasculitis with corticosteroids. There is currently a paucity of literature describing cholestatic hepatitis secondary to large vessel vasculitides, and more awareness of these diseases as an etiology for unexplained elevation of liver enzymes is warranted, to assist in early diagnosis and treatment of GCA/PMR.

## Case 1

A 62-year-old male presented with a three-week history of fatigue, fever and abnormal liver enzymes. The patient was diagnosed with rheumatoid arthritis eight months prior to presentation, which manifested with symptoms of decreased range of motion in his hips and swollen proximal interphalangeal joints. He was initially treated with prednisone and leflunomide but was transitioned to etanercept once weekly, with withdrawal of prednisone. After the seventh dose of etanercept, the patient began experiencing fevers up to 102°F, general malaise, diaphoresis and a dull bilateral headache. He denied any visual changes, temporal tenderness or jaw claudication. The patient’s medications at that time also included dexlansoprazole and atorvastatin. He had no significant family history and was a social drinker. Physical examination was significant for a mild increase in liver span. There was no abdominal tenderness, jaundice, temporal tenderness or joint swelling.

Laboratory tests revealed alkaline phosphatase of 1017 U/L, aspartate transaminase (AST) of 306 U/L and alanine transaminase (ALT) of 344 U/L. Complete blood cell count, coagulation panel and basic metabolic panels were within normal limits, whereas erythrocyte sedimentation rate (ESR) was 97 mm/h, and C-reactive protein (CRP) was 16.3 mg/dL. Antinuclear antibody (ANA), anti-smooth muscle antibody (ASMA), anti-mitochondrial antibody (AMA), *Cytomegalovirus* polymerase chain reaction and viral hepatitis A/B/C serologies were all negative. Abdominal ultrasound showed normal liver parenchyma, patent hepatic arteries and veins and portal veins with normal direction of flow. Computed tomography (CT) scan of the abdomen and pelvis was unremarkable. There was no intrahepatic or extrahepatic ductal dilatation. A liver core biopsy showed minimal nonspecific focal portal and lobular inflammation.

Initially, the patient was thought to have drug-induced liver injury secondary to etanercept. The patient was started on prednisone 20 mg for treatment of drug-induced liver injury, which resulted in improvement of his symptoms and normalization of his liver enzymes. During a trial of a prednisone taper, however, the patient’s AST, ALT, and alkaline phosphatase rose.

**Figure 1.**
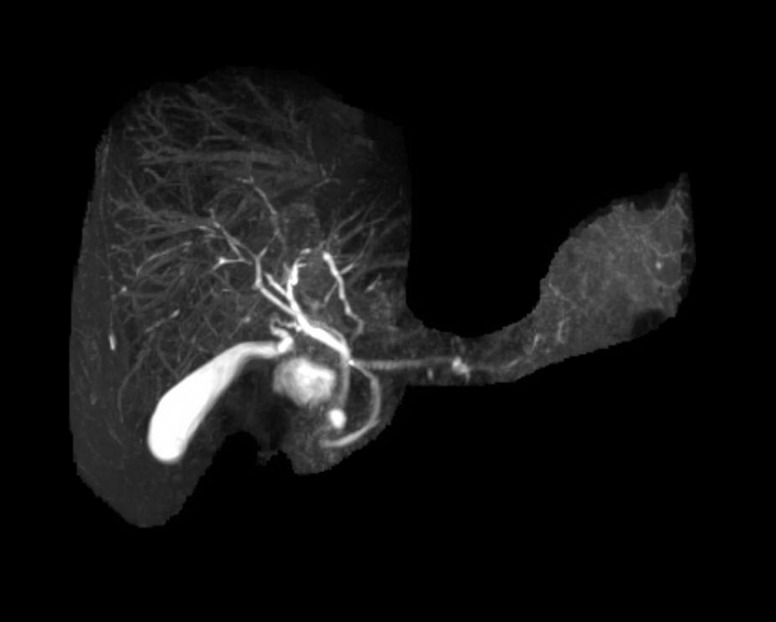
MRCP demonstrates significant irregularity of the intrahepatic bile ducts with areas of narrowing and beading-type appearance suggestive of cholangitis.

**Figure 2.**
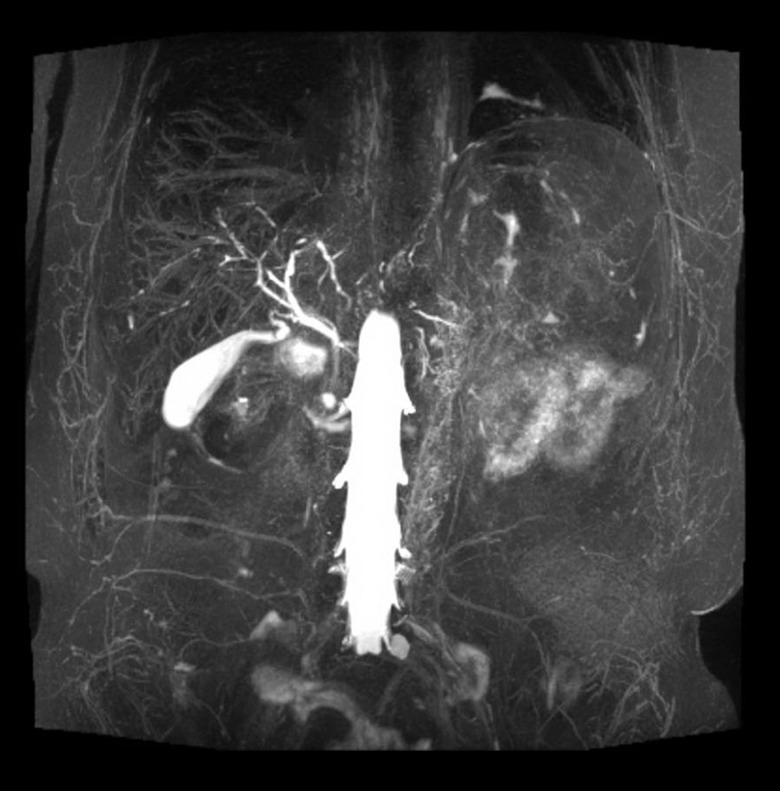
The aortic wall is diffusely thickened. There is marked edema as noted on T2 STIR sequences as well as increase in signal on high B-value consistent with edema. On post-contrast administration, there is interval increase in the enhancement of the aortic wall suggestive of active vasculitis.

Magnetic resonance angiogram (MRA) of the abdomen and pelvis and magnetic resonance cholangiogram (MRCP) demonstrated an abnormal aortic wall with edema and enhancement consistent with vasculitis as well as abnormal intrahepatic ducts with areas of stenosis suggestive of cholangitis (Figures 1 and 2). In correlating the radiologic findings with his physical symptoms of fatigue, fever, joint pain and elevated ESR, the patient was diagnosed with giant cell arteritis. Prednisone 60 mg was started, and his liver enzymes normalized again two weeks later and remained within normal limits (Table 1). Follow-up MRCP, after liver enzymes normalized, revealed no evidence of ductal abnormality.

**Table 1.** Lab test results for Case 1.

Time	T Bili (mg/dL)	AST (U/L)	ALT (U/L)	ALP (U/L)	GGT (U/L)	HB (g/dL)	HCT (%)	PLT (×10^9^/L)	WBC (×10^9^/L)	ESR (mm/h)	CRP (mg/dL)
Six months before onset of symptoms	0.5	23	20	72	34	–	–	–	–	–	–
Three weeks after onset of symptoms	0.5	306	344	1017	215	12.6	37.6	446	7.3	97	16.3
Four weeks after starting treatment (prednisone 20mg)	0.5	11	30	156	99	12.1	37.8	384	8.6	100	31
Five weeks after starting treatment (prednisone 20mg)	0.5	13	22	126	72	11.5	35.5	367	7.8	102	39
Four weeks after stopping treatment	0.5	138	173	557	293	11.7	35.8	469	8.1	>130	146
Two weeks after restarting treatment (prednisone 60 mg)	0.3	12	18	162	75	13.1	41	388	16.5	36	25
Four weeks after restarting treatment (prednisone 60mg)	0.4	15	23	103	54	13.8	42.9	248	10	22	4.4
Eight weeks after restarting treatment (prednisone 40mg)	0.3	13	17	82	34	13.7	41.7	324	11.3	–	–

ALP = alkaline phosphates; ALT = alanine transaminase; AST = aspartate transaminase; CRP = C-reactive protein; ESR = erythrocyte sedimentation rate; GGT = gamma-glutamyl transpeptidase; HB = hemoglobin; HCT = hematocrit; PLT = platelet; T Bili = total bilirubin; WBC = white blood cell

## Case 2

A 74-year-old male presented to the emergency room with a two-to-three week history of increasing lethargy, intermittent fever and anorexia. Prior to his visit to the emergency room, he was seen by his general practitioner; due to muscle pain, he was treated for one week with celecoxib without any significant improvement of his symptoms. The patient did not have any abdominal complaints. The patient had a history of hypertension and hyperlipidemia and was treated for several years with losartan and simvastatin. He did not have any history of liver disease and had very modest alcohol consumption. Labs were significant for AST 222 U/L, ALT 190 U/L, alkaline phosphatase 411 U/L, gamma-glutamyl transpeptidase (GGT) 231 U/L, normal bilirubin, serum albumin 2.6 g/dL and normal international normalized ratio. The patient had a hemoglobin of 10.2 g/dL, platelet count of 600 × 10^3^/µL and ESR of 100 mm/h. Serologies for hepatitis A, B, C, E, *Cytomegalovirus*, and Epstein-Barr virus as well as AMA, ANA and ASMA were negative. He was hospitalized for three days for work-up of a possible malignancy since this was considered the most likely diagnosis. CT scan of the abdomen was however normal.

The initial diagnosis was drug-induced liver injury, and both simvastatin and losartan were discontinued. The patient’s liver tests remained elevated one week later at a follow-up appointment at the outpatient clinic. Detailed history-taking elicited additional symptoms of muscle pain, mostly in the thighs and shoulders as well as difficulty standing up from a chair and lifting his arms. History of these characteristic symptoms of PMR had not been obtained during the initial hospitalization. On exam, the patient’s proximal muscles were very tender, although he was not tender over the temporal arteries and did not have visual symptoms.

Due to his symptoms and laboratory parameters, he was clinically diagnosed with PMR. He was started on prednisolone 40 mg for one week with near-complete resolution of his myalgia. One week later, his alkaline phosphatase decreased from 411 U/L to 169 U/L, and ESR decreased from 100 to 30 mm/h. After four weeks of treatment with prednisolone 40 mg/d for 2 weeks and 20 mg/d for 2 weeks, his liver enzymes, platelets and albumin were normal. His ESR was also normal at 10 mm/h.

## Discussion

We present two patients with severe elevation of alkaline phosphatase secondary to GCA/PMR. Although elevation of hepatic aminotransferases has been described in those with GCA/PMR [5], this association has been depicted only in a small number of case reports [3,6–11]. In most instances, patients present with constitutional symptoms of fever, weight loss, night sweats and anorexia with elevations in ESR and CRP. When liver tests are abnormal, a rise in serum alkaline phosphatase is consistently seen [12], while hyperbilirubinemia is not typically seen, and serum AST and ALT levels may be normal or elevated [7].

Histological changes of the liver in GCA/PMR are inconsistent and nonspecific. These may include normal histology [1], moderate bile stasis [2], fatty degeneration [3] and nonspecific portal tract inflammatory infiltration [4]. Although the underlying pathophysiology remains to be elucidated, it is proposed that the abnormal liver enzymes result from inflammation of the hepatic artery [13]. In one report, biopsy of the hepatic and temporal arteries in the same patient demonstrated lymphocytic inflammatory infiltrates and numerous giant cells, which are typical of giant cell arteritis [9]. The elevated alkaline phosphatase often associated with GCA/PMR is likely a result of injury to bile duct epithelial cells due to adjacent arteritis.

In the first case we presented, MRA showed increased arterial wall enhancement and a thickened aortic wall. There was also irregularity of the intrahepatic bile ducts with a beading-type appearance consistent with cholangitis secondary to arteritis of hepatic vasculature. Similarly, in a case reported by Prabhavalkar *et al*, a 60-year-old female was found to have elevated ESR, alkaline phosphatase and AST [6]. A whole body positron emission tomography scan demonstrated increased metabolic activity within the central large vessels, thoracic and abdominal aorta, and proximal carotids and subclavian arteries consistent with a large vessel vasculitis. A temporal artery biopsy confirmed the diagnosis of GCA. The severity of the clinical manifestations and abnormal liver function tests appear to correspond to the severity of the arteritis. In most cases, including the two presented above, there is complete resolution of abnormal liver enzymes after adequate corticosteroid treatment [7].

In patients with cholestatic liver enzymes secondary to large vessel vasculitis such as GCA/PMR, we recommend immediate referral to a rheumatologist to determine proper dosing of corticosteroid-based therapy [14]. As the abnormal liver enzymes correlate with disease activity, it is expected that control of the underlying vasculitis will lead to improvement in aminotransferase levels. The patient should be closely followed by a rheumatologist to adjust corticosteroid dosing, with the goal of achieving disease remission. Liver biopsy is not necessary if the patient responds appropriately to treatment.

Given the seriousness of untreated GCA/PMR with potential complications of blindness or ischemic cerebrovascular accident, it is crucial to recognize the possibility of this condition as a cause of liver enzyme elevation. Therefore, in patients aged >50 years with elevated liver enzymes, persistent fever and elevated ESR, consideration should be given to diagnosis and treatment of large vessel vasculitis.

*Conflict of interest statement:* none declare.
